# Supplementary material for: MK8617 inhibits M1 macrophage polarization and inflammation via the HIF-1α/GYS1/UDPG/P2Y14 pathway
Source: PeerJ. 2023 Jun 30;11:e15591. doi: 10.7717/peerj.15591 (PMC10317019; doi:10.7717/peerj.15591)
Supplement: Supplemental Information 1 — qPCR reaction conditions, primer sequence and shRNA sequence used in the research. [file peerj-11-15591-s001.docx]

Supplementary Table 1.qPCR reaction conditions

| Stage 1 | Pre-mutability | Rep:1 | 95℃ | 30sec |
| --- | --- | --- | --- | --- |
| Stage 2 | Circular reaction | Reps:40 | 95℃ | 3-10sec |
|  |  |  | 60℃ | 10-30sec |
| Stage 3 | Melting Curve | Rep:1 | 95℃ | 15sec |
|  |  |  | 60℃ | 60sec |
|  |  |  | 95℃ | 15sec |

Supplementary Table 2.Primer sequence

| Gene | Primer | Primer sequence. |
| --- | --- | --- |
| Mouse TNF-α | F | GGTGCCTATGTCTCAGCCTCTT |
|  | R | GCCATAGAACTGATGAGAGGGAG |
| Mouse IL-1β | F | GCAACTGTTCCTGAACTCAACT |
|  | R | ATCTTTTGGGGTCCGTCAACT |
| Mouse IL-6 | F | TAGTCCTTCCTACCCCAATTTCC |
|  | R | TTGGTCCTTAGCCACTCCTTC |
| Mouse HIF-1α | F | TTTGCCCAGGGGACTAAAGC |
|  | R | TGTGCAGTATTGTAGCCACG |
| Mouse GYS1 | F | CACAGAACGGTTGTCGGACTTG |
|  | R | AGGTGAAGTGGTCTGGAAAGGC |
| Mouse P2Y14 | F | ACCTCCGTCAAGAGGAAGTCCA |
|  | R | GCTGTAGTGACCTTCCGTCTGA |
| Mouse Pgm1 | F | AGCCAATGACCCAGATGCTGAC |
|  | R | TCCAGGAAGTGAAGAGCCACCA |
| Mouse Ugp2 | F | CTGATGAACCCACCCAATGGGA |
|  | R | GAGCGATTTCCACCAGTCTCAG |
| Mouse β-actin | F | GGCTGTATTCCCCTCCATCG |
|  | R | CCAGTTGGTAACAATGCCATGT |
| Human TNF-α | F | CTCTTCTGCCTGCTGCACTTTG |
|  | R | ATGGGCTACAGGCTTGTCACTC |
| Human IL-1β | F | CCACAGACCTTCCAGGAGAATG |
|  | R | GTGCAGTTCAGTGATCGTACAGG |
| Human IL-6 | F | ACTCACCTCTTCAGAACGAATTG |
|  | R | CCATCTTTGGAAGGTTCAGGTTG |
| Human β-actin | F | CACCATTGGCAATGAGCGGTTC |
|  | R | AGGTCTTTGCGGATGTCCACGT |

Supplementary Table 3.shRNA sequence

| Mouse HIF-1α | shRNA#1 | GCTCATCCAAGGAGCCTTAAC |
| --- | --- | --- |
|  | shRNA#2 | GCTCACCATCAGTTATTTACG |
|  | shRNA#3 | GCAGGAATTGGAACATTATTG |
| Mouse GYS1 | shRNA#1 | CCTGGAGAATTTCAATGTA |
|  | shRNA#2 | GCACCTGGACTTC AACCTA |
|  | shRNA#3 | GCAGCTGCCCGCCCGATTC |
