# Supplementary figures and images for: MK8617 inhibits M1 macrophage polarization and inflammation via the HIF-1α/GYS1/UDPG/P2Y14 pathway
Source: PeerJ. 2023 Jun 30;11:e15591. doi: 10.7717/peerj.15591 (PMC10317019; doi:10.7717/peerj.15591)

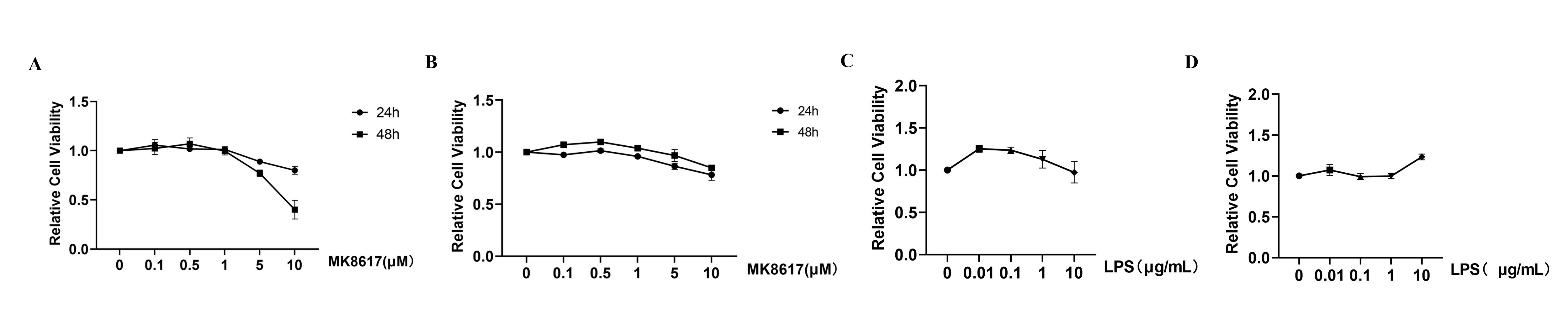

Supplement: Supplemental Information 2 — (A) Cell viability of MK8617 on RAW264.7 macrophages. (B) Cell viability of MK8617 on PMA incubated THP-1 cells. (C) Cell viability of LPS on RAW264.7 macrophages. (D) Cell viability of LPS on PMA incubated THP-1 cells. Data are mean ± SEM (n = 3) [file peerj-11-15591-s002.png]

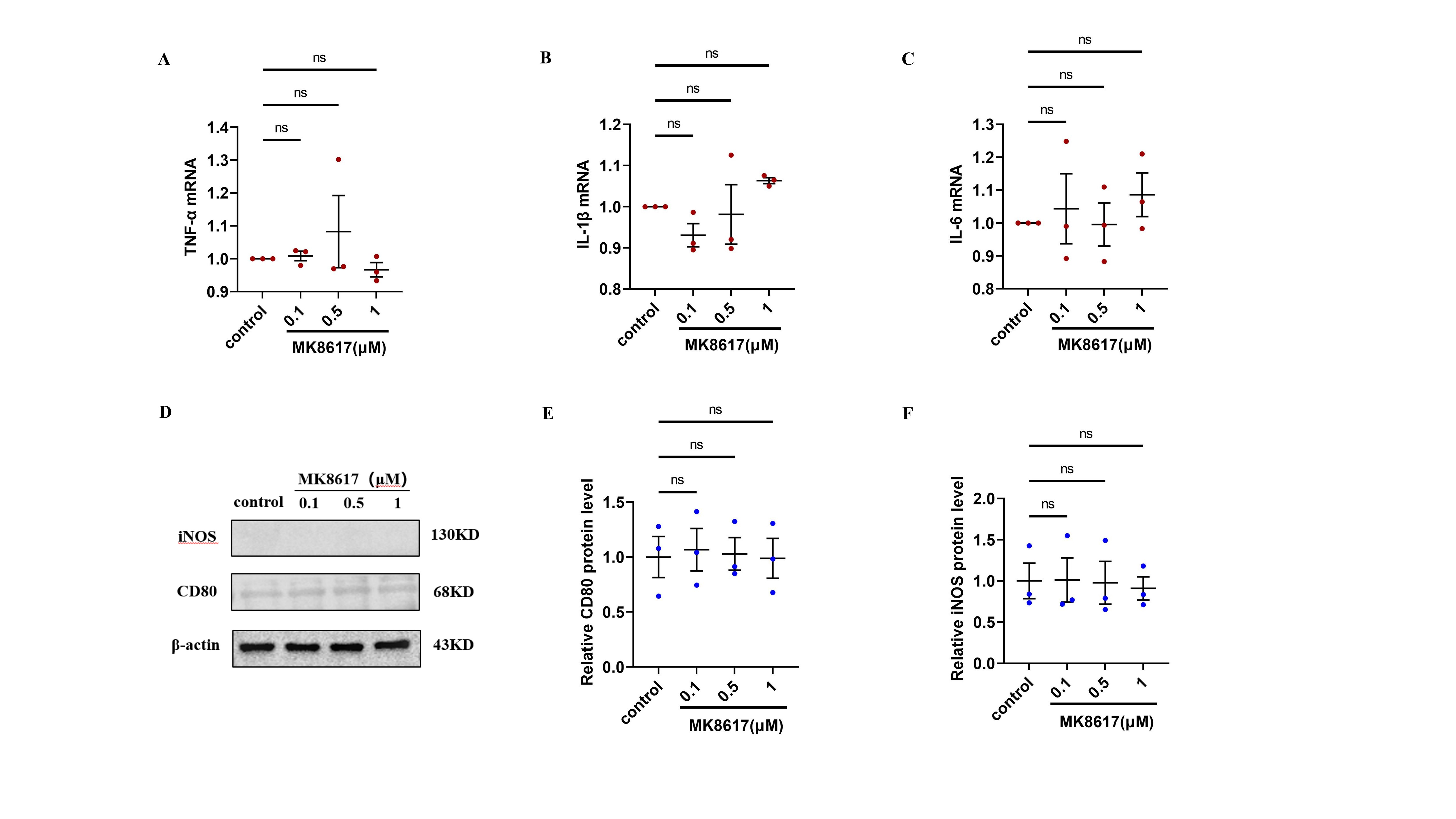

Supplement: Supplemental Information 3 — mRNA levels of inflammatory cytokines were determined using qRT-PCR: (A) TNF-α (B) IL-β (C) IL-6. (D–F) WB was used to determine CD80 and iNOS expression. Data are mean ± SEM (n = 3). “ns” indicates no significance (p > 0.05). [file peerj-11-15591-s003.png]

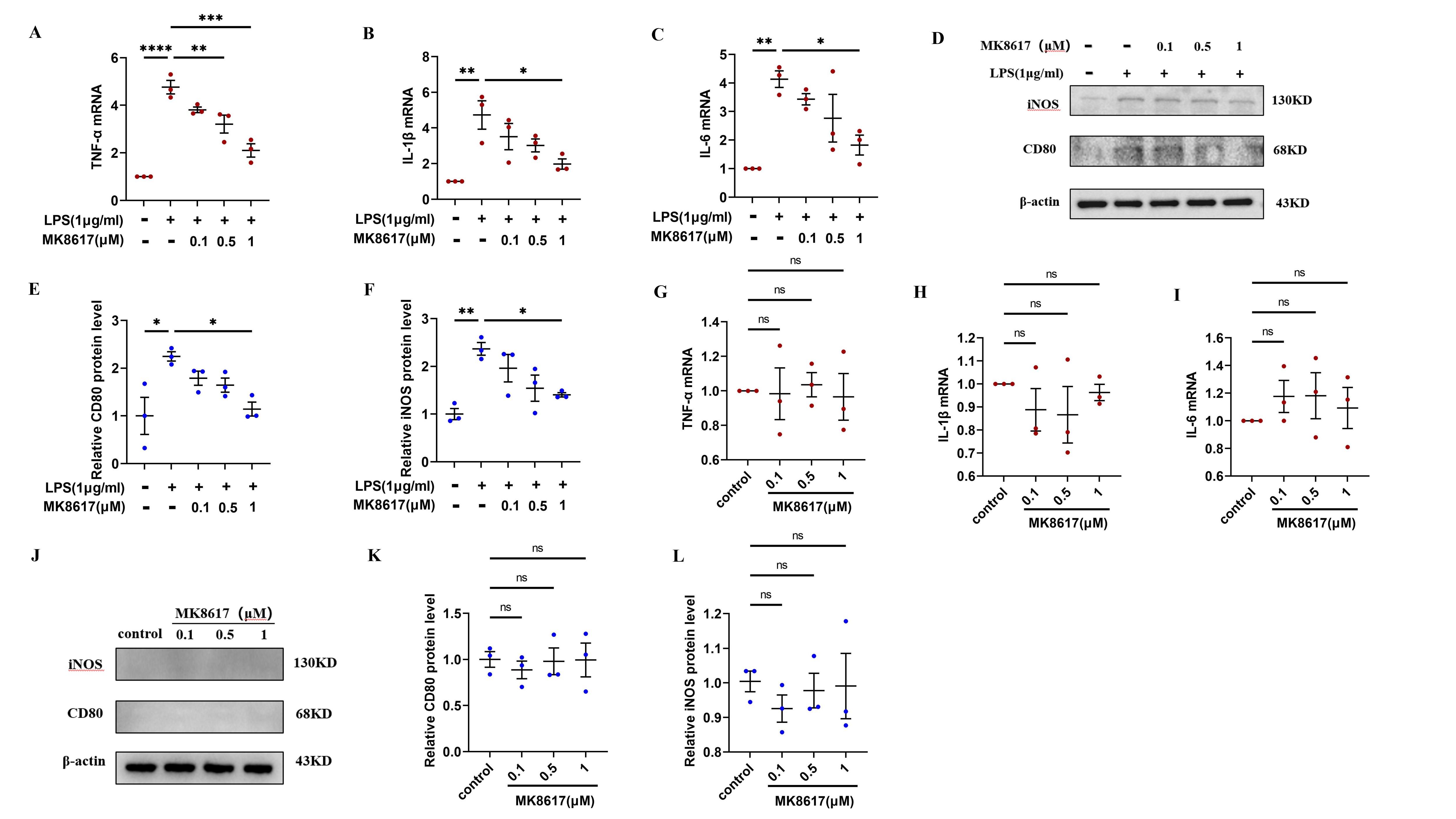

Supplement: Supplemental Information 4 — mRNA levels of inflammatory cytokines were determined using qRT-PCR: (A) TNF-α (B) IL-β (C) IL-6. (D–F) WB was used to determine CD80 and iNOS expression. PMA incubated THP-1 cells were treated with MK8617 alone and then inflammatory indicators were detected. (G–I) qRT-PCR results of TNF-α, IL-β, IL-6. (J–L) WB results of CD80 and iNOS. Data are mean ± SEM (n = 3). *p < 0.05, **p < 0.01, ***p < 0.001, ****p < 0.0001. [file peerj-11-15591-s004.png]

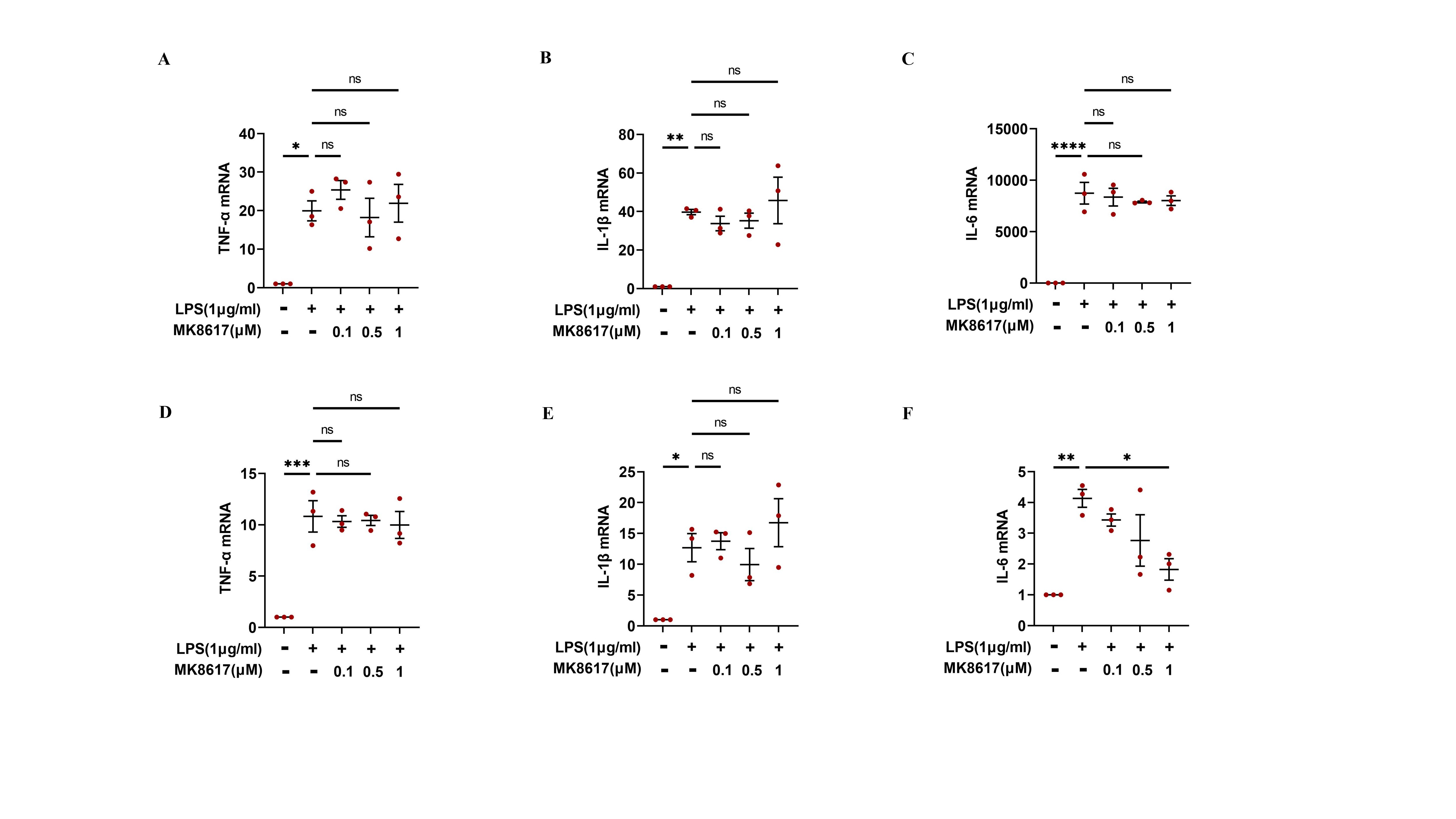

Supplement: Supplemental Information 5 — RAW264.7 cells were stimulated with LPS to induce inflammation and then treated with MK8617 for 24 h. mRNA levels of inflammatory cytokines were determined using qRT-PCR: (A) TNF-α (B) IL-β (C) IL-6. PMA incubated THP-1 cells were treated with the same method as RAW264.7 cells. mRNA levels of inflammatory cytokines were determined using qRT-PCR: (D) TNF-α (E) IL-β (F) IL-6. Data are mean ± SEM (n = 3). *p < 0.05, **p < 0.01, ***p < 0.001, ****p < 0.0001. [file peerj-11-15591-s005.png]

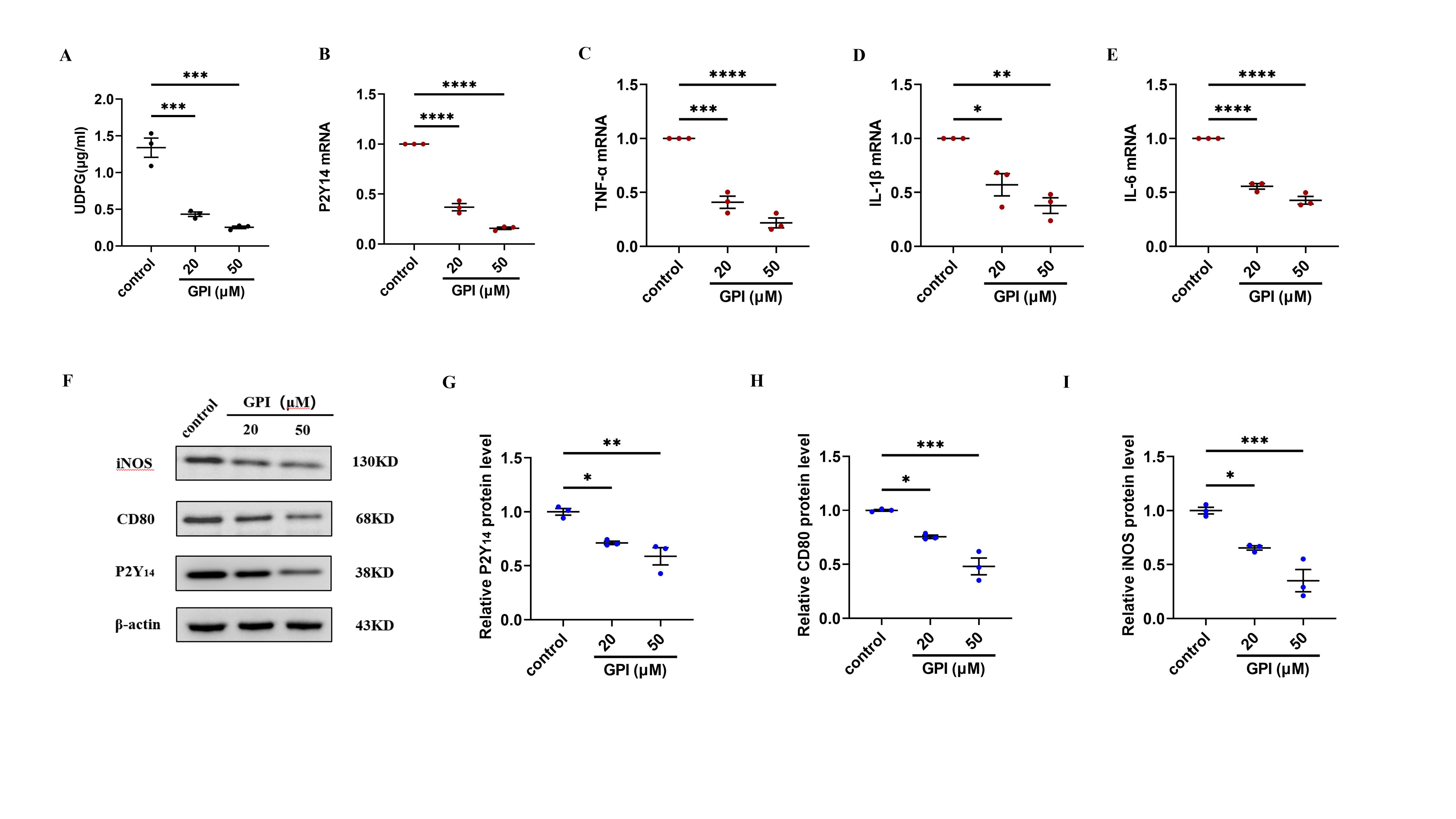

Supplement: Supplemental Information 6 — RAW264.7 cells were pretreated for 30 min with vehicle (control) or GPI (20 or 50 μM) prior to stimulation with LPS for 24 h. (A) Supernatant was collected to determine UDPG content by ELISA. (B–E) qRT-PCR results of P2Y14, TNF-α, IL-β, IL-6. (F–I) WB results of P2Y14, CD80 and iNOS. Data are mean ± SEM (n = 3). *p < 0.05, **p < 0.01, ***p < 0.001, ****p < 0.0001. [file peerj-11-15591-s006.png]

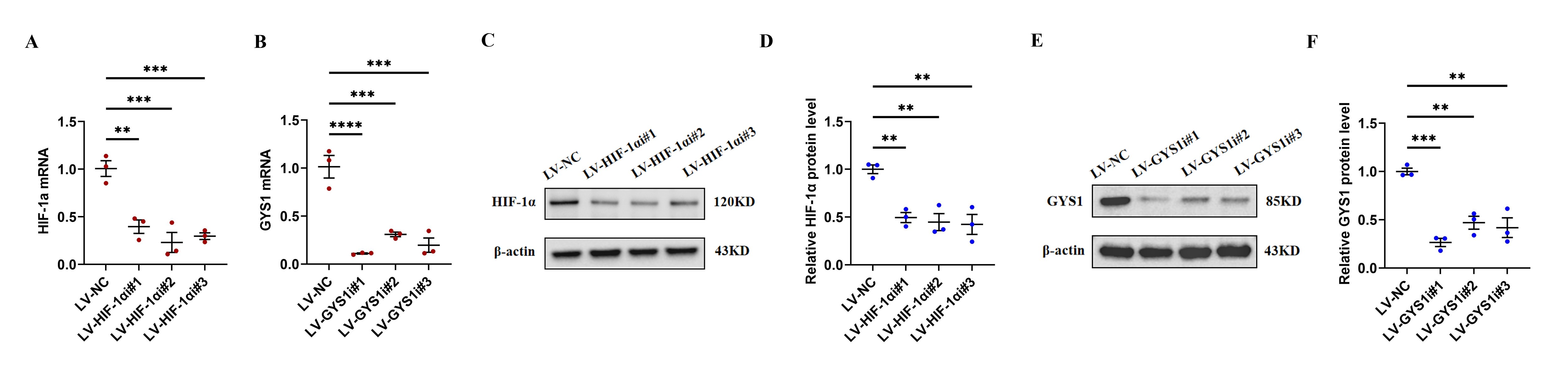

Supplement: Supplemental Information 7 — (A, B) qRT-PCR results of HIF-1α and GYS1. (C–F) WB results of HIF-1α and GYS1. Data are mean ± SEM (n = 3). *p < 0.05, **p < 0.01, ***p < 0.001, ****p < 0.0001. [file peerj-11-15591-s007.png]

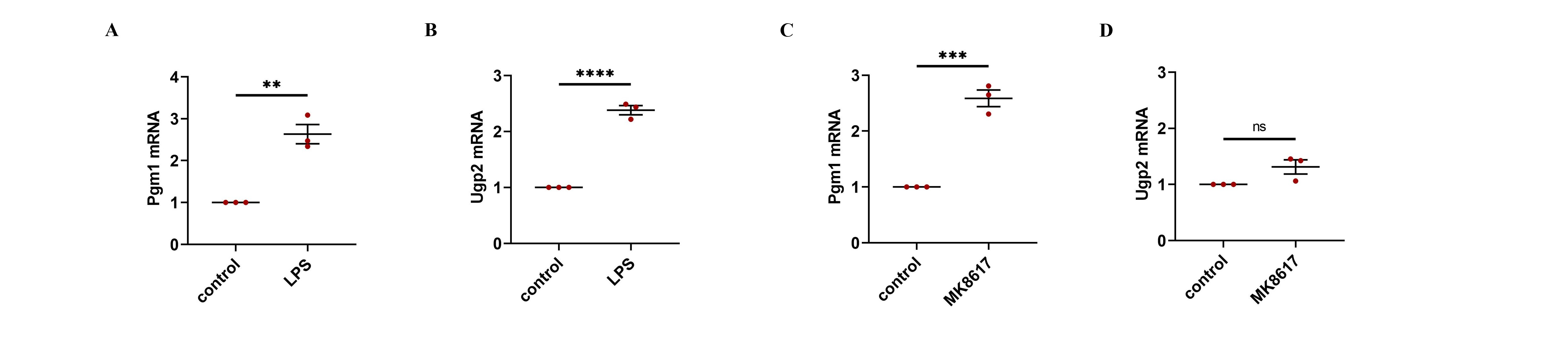

Supplement: Supplemental Information 8 — (A–D) mRNA levels of Pgm1 and Ugp2 were determined using qRT-PCR. Data are mean ± SEM (n = 3). *p < 0.05, **p < 0.01, ***p < 0.001, ****p < 0.0001. [file peerj-11-15591-s008.png]
